# Identification of frailty heterogeneity and its transition trajectories in patients with chronic heart failure: a latent transition analysis

**DOI:** 10.3389/fmed.2026.1862183

**Published:** 2026-07-02

**Authors:** Shun Zhang, Yuqin Huang, Xiaohan Zhai, Hongrong Huang, Ming Hou, Yan Wang

**Affiliations:** 1 Department of Nursing, Shihezi University School of Medicine, Shihezi, China; 2 The First Affiliated Hospital of Shihezi University, Shihezi, China; 3 People’s Hospital of Xinjiang Uygur Autonomous Region, Ürümqi, China

**Keywords:** chronic heart failure, frailty, latent profile analysis, latent transition analysis, risk factors

## Abstract

**Objective:**

To explore the transitions of potential frailty subtypes and their influencing factors in patients with chronic heart failure (CHF), and to provide evidence for the development of targeted frailty intervention strategies.

**Methods:**

A longitudinal follow-up study was conducted. A total of 324 hospitalized CHF patients were consecutively and conveniently enrolled from the cardiovascular departments of two general hospitals in Xinjiang, China, between December 2024 and April 2025. Baseline assessments were performed on the second day of hospitalization (T1), and follow-up surveys were carried out at 3 months (T2) and 6 months (T3) after discharge. Latent Profile Analysis (LPA) was adopted to identify frailty subtypes, Latent Transition Analysis (LTA) was used to analyze the dynamic transition characteristics of different frailty subtypes, and multivariate logistic regression analysis was applied to explore the factors associated with subtype transitions.

**Results:**

Three relatively stable frailty subtypes were identified among CHF patients, namely healthy type with low frailty, social-dominant type with moderate frailty, and psychological-dominant type with high frailty. The social-dominant moderate frailty subtype was mainly characterized by insufficient social support, while the psychological-dominant high frailty subtype presented with heavier psychological burden and more severe functional impairment. Dynamic transitions were observed across all subtypes during follow-up. The moderate frailty subtype had the lowest stability and was prone to progression toward the high frailty subtype. The transition rate from moderate frailty to high frailty was 24.4% during the T1→T2 period, which further increased to 34.0% in the T2→T3 period. Age, medical insurance status, New York Heart Association (NYHA) functional classification, N-terminal pro-brain natriuretic peptide (NT-proBNP), estimated glomerular filtration rate (eGFR), insomnia, depression and home volume management level were significant influencing factors for frailty subtype transitions (*P* < 0.05).

**Conclusion:**

Frailty among CHF patients is not a single static condition, but presents obvious heterogeneity and dynamism. Patients with social-dominant moderate frailty represent a high-risk group for frailty deterioration, and those with psychological-dominant high frailty suffer from severe psychological burden and functional impairment. Stratified management targeting different frailty subtypes, with special attention to elderly patients, patients without medical insurance, those with poor cardiac function, renal impairment, insomnia and inadequate home volume management, may help reduce the risk of frailty progression and improve long-term health outcomes.

## 1 Introduction

Chronic heart failure (CHF) represents the end-stage progression of various cardiovascular diseases and is one of the leading causes of readmission and mortality among elderly patients. It has been reported (1) that the number of heart failure (HF) cases in China will reach 22.7 million by 2035, with the cumulative economic burden accounting for 0.26% of the gross domestic product (GDP). Frailty (2) refers to a vulnerable state resulting from reduced physiological reserve of multiple body systems and diminished stress resistance. Frailty in patients with heart failure (3) is defined as a multidimensional, dynamic and potentially reversible condition. Though correlated with age, it is not entirely determined by aging. This condition renders HF patients more susceptible to stressors and substantially elevates the risk of adverse clinical outcomes. Comorbidity of frailty among CHF patients has become a prevalent and critical clinical issue worldwide. A meta-analysis revealed (4) that the overall pooled prevalence of frailty among heart failure patients globally is 44.5%, with a slightly higher rate of 47.4% for multidimensional frailty. Relevant domestic data (5) indicate that the prevalence of frailty among hospitalized HF patients in China is as high as 68.7%, markedly higher than that in the general elderly population. Accordingly, frailty has become a syndrome that cannot be overlooked in the disease management and prognosis assessment of patients with CHF in China.

In recent years, research on frailty has been increasingly advanced. As a multidimensional syndrome, frailty presents prominent heterogeneity (6). Individuals differ greatly in the degree of impairment in physical, psychological and social functioning, implying the existence of distinct potential subtypes within the frail population. Latent Profile Analysis (LPA) (7), an individual-centered statistical approach, can classify subjects based on multidimensional indicators to identify latent heterogeneity in a population. It provides a novel analytical perspective for exploring differentiated characteristics and distribution patterns among frail individuals. In recent years, LPA has been widely adopted in heterogeneity research across various populations (8, 9), yet its application in identifying multidimensional frailty subtypes among CHF patients remains limited. Previous studies have demonstrated (6, 10) that frailty is reversible with targeted interventions, and individuals may transit between different frailty states. Accordingly, cross-sectional analysis alone is insufficient to fully clarify the developmental trajectories and outcomes of frailty in CHF patients. Although conventional trajectory models (11) can categorize patients into distinct frailty trajectory groups, they generally assume relatively stable status after grouping, and thus fail to reflect the dynamic shifts and mutual transitions of frailty during disease progression. A longitudinal study conducted by Gill et al. (12) among community-dwelling older adults confirmed bidirectional transitions across robust, pre-frail and frail states. Nevertheless, this study described state transitions merely based on predefined frailty grades, and was unable to identify latent heterogeneous subgroups within the population as well as their transition patterns.

Latent transition analysis (LTA) (13), an extension of LPA, enables further analysis of individual transitions between distinct latent states using longitudinal data. It can not only estimate transition probabilities across different states, but also incorporate covariates to explore key factors affecting such transitions. For patients with chronic heart failure (CHF), identifying frailty subtypes and their transition patterns facilitates early detection of individuals at high risk of frailty progression, and helps clinicians clarify the main vulnerable dimensions of each patient, thereby supporting the delivery of individualized nursing and clinical interventions. Existing studies (14, 15) have indicated that the first 3 months after hospital discharge constitute a critical period for disease recovery and treatment adjustment among heart failure patients, while the period from 3 to 6 months post-discharge is characterized by relative clinical stability. Therefore, follow-up assessments were scheduled at 3 and 6 months to capture dynamic changes in patients’ frailty status across different stages. This study adopted LTA to explore transition patterns among latent frailty subtypes in CHF patients and analyze associated influencing factors, aiming to provide evidence for identifying high-risk populations and implementing targeted interventions in future clinical practice.

## 2 Materials and methods

### 2.1 Study design

Convenience sampling was adopted in this study. Between November 2024 and April 2025, consecutive inpatients diagnosed with chronic heart failure were enrolled from the cardiovascular departments of two general hospitals in Xinjiang, China, to minimize selection bias. Evidence (16, 17) has shown that dynamic changes in frailty can occur within weeks to months. Accordingly, the baseline assessment (T1) was conducted during hospitalization, and follow-up surveys were performed at 3 months (T2) and 6 months (T3) after discharge. This study was approved by the hospital Research Ethics Committee (Approval No. KJ202452402).

### 2.2 Inclusion and exclusion criteria

Inclusion criteria were as follows: (1) diagnosis of chronic heart failure according to the 2024 Chinese guidelines for chronic heart failure (18), covering heart failure with reduced ejection fraction (HFrEF), heart failure with mildly reduced ejection fraction (HFmrEF), and heart failure with preserved ejection fraction (HFpEF); (2) New York Heart Association (NYHA) functional class IIHFmr (19); and (3) provision of informed consent with adequate reading comprehension and communication ability.

Exclusion criteria were: (1) unwillingness to participate in follow-up for personal reasons; (2) presence of severe acute cardiovascular events or other life-threatening acute illnesses; and (3) coexistence of malignant tumors or other end-stage diseases. Withdrawal criteria were: (1) incomplete follow-up data or loss to follow-up.

### 2.3 Sample size calculation

The sample size was calculated based on the requirement for logistic regression analysis, namely that the sample size should be 5−10 times the number of independent variables. This study included 15 independent variables. With an estimated maximum follow-up loss rate of 20%, the required sample size was calculated to be between 94 and 188 cases. Existing evidence indicates that a sample size of no < 300 is necessary to ensure robust and reliable results of Latent Profile Analysis (7). Ultimately, a total of 324 participants were enrolled in this study.

### 2.4 Data collection and quality control

Before data collection, all investigators received standardized training on the study protocol, questionnaire administration procedures, standardized instructions, and telephone follow-up techniques to ensure consistency throughout the study. After obtaining written informed consent, paper-based questionnaires were administered to eligible participants on the second day of hospitalization. For participants who had difficulty completing the questionnaires independently, trained researchers provided assistance when necessary. Upon questionnaire completion, participants’ contact information was recorded and the follow-up schedule was explained. Completed questionnaires were reviewed according to predefined criteria, and invalid questionnaires were excluded. Clinical data, including LVEF and NYHA functional class, were extracted from the hospital electronic medical record system. Follow-up assessments at 3 months (T2) and 6 months (T3) after discharge were conducted via telephone interviews by the same trained researchers using standardized procedures. Responses were recorded item by item during each interview. To ensure data accuracy, all data were independently entered by two researchers and cross-checked for consistency. Any discrepancies were resolved by referring to the original questionnaires and source documents.

### 2.5 Survey instruments

#### 2.5.1 Demographic questionnaire

A self-designed questionnaire was developed based on extensive literature review. The collected indicators encompassed gender, age, marital status, medical insurance status, educational level, disease duration, comorbid chronic diseases, estimated glomerular filtration rate (eGFR), left ventricular ejection fraction (LVEF), and New York Heart Association (NYHA) functional classification.

#### 2.5.2 Tilburg frailty indicator (TFI)

The TFI was developed by Gobbens et al. (20), consisting of 15 items across three dimensions. The social dimension covers living alone, social relationships and social support; the psychological dimension includes memory, depression, anxiety and coping ability; the physical dimension involves general physical health, unintentional weight loss, walking difficulty, visual impairment, hearing problems, balance, hand weakness and fatigue. A total score of 5 or above indicates frailty, with higher scores representing more severe frailty. The Cronbach’s α coefficient of the Chinese version of the TFI is 0.686 (21), which is within an acceptable range. Although its internal consistency differs from that of the Polish version (22), this multidimensional frailty assessment tool has been widely used in frailty-related studies among the elderly and patients with chronic diseases in China, with satisfactory construct validity, predictive validity and clinical applicability (23).

#### 2.5.3 Insomnia severity index (ISI)

The Insomnia Severity Index (ISI) is a widely used international tool for the subjective assessment of insomnia. Developed by Morin et al. (24), it is designed to evaluate the severity of an individual’s insomnia symptoms over the past 2 weeks and their impact on daytime functioning. The ISI consists of 7 items, each scored on a 5-point scale ranging from 0 to 4, with a total score ranging from 0 to 28. A higher score indicates more severe insomnia. The Cronbach’s alpha coefficient for this scale is 0.856.

#### 2.5.4 Patient Health Questionnaire (PHQ-9)

Developed by Kroenke et al. (25) to quantify depression, this scale consists of 9 items, each scored on a scale from 0 (not at all) to 3 (almost every day), with a total score ranging from 0 to 27. Scoring criteria: 0–4 points indicate no depression; 5–9 points indicate mild depression; 10–14 points indicate moderate depression; 15–19 points indicate moderate-to-severe depression; and 20–27 points indicate severe depression. The Cronbach’s alpha coefficient for this scale is 0.716.

#### 2.5.5 Self-assessment scale for home volume management in patients with chronic heart failure

Developed by Chinese scholars Ye et al. (26), this scale comprises four dimensions: self-care assessment, self-care maintenance, self-care management, and self-care confidence, with a total of 27 items. The scale uses a 5-point Likert scale, with a total score ranging from 27 to 135; a higher score indicates a stronger level of volume management. The Cronbach’s alpha coefficient for this scale is 0.894.

### 2.6 Statistical analysis

Data analyses were performed using SPSS 27.0 and Mplus for latent profile analysis (LPA) and latent transition analysis (LTA). Categorical variables were described by frequencies and percentages. Normally distributed continuous variables were presented as mean ± standard deviation (SD). For missing data with a low missing rate and under the assumption of missing completely at random, multiple imputation was applied to minimize bias caused by data loss. Since insomnia, depression, home volume management and frailty were all assessed via self-report, Harman’s single-factor test was used to examine potential common method bias. All items of scales at each time point were included in the unrotated exploratory factor analysis. The average scores of the three frailty dimensions were taken as observed variables to establish the LPA model. Model fit was evaluated using the Akaike Information Criterion (AIC), Bayesian Information Criterion (BIC), sample-adjusted Bayesian Information Criterion (aBIC), and Entropy. The Bootstrap Likelihood Ratio Test (BLRT) and Lo-Mendell-Rubin Adjusted Likelihood Ratio Test (LMR) were also adopted for model selection. LTA was conducted to calculate transition probabilities across different time points and illustrate the temporal trends and transition patterns of latent subgroups. Multivariate logistic regression was performed to explore the effects of demographic variables, depression, insomnia and home volume management on transitions between frailty subgroups. Latent transition classes derived from LTA were set as dependent variables, and participants who remained in their original subgroups served as the reference group. All independent variables were entered into the regression model to calculate odds ratios (OR). An OR > 1 indicated a higher probability of transitioning to the target subgroup, while an OR < 1 suggested a lower probability. The significance level was set at α = 0.05.

## 3 Results

### 3.1 Common method bias assessment

The results showed that the variance explained by the first principal component was 13.88% at T1, 14.4% at T2, and 14.77% at T3. All values were substantially lower than the critical threshold of 40%, indicating no serious common method bias in the data across the three time points.

### 3.2 Demographic characteristics of patients with CHF

A total of 338 questionnaires were distributed, and all participants completed the baseline assessment. After excluding four invalid questionnaires, 334 patients with CHF were included in the final analysis. During follow-up, 329 participants completed the 3-month assessment (T2), with five losses to follow-up (two could not be contacted and three voluntarily withdrew). At the 6-month assessment (T3), 324 participants completed follow-up, with an additional five losses (two could not be contacted, two withdrew due to disease progression, and one died). The overall follow-up completion rate was 97.0%. The follow-up flow diagram is presented in Supplementary Figure 1. The mean age of the participants was 64.1 ± 11.97 years. Other demographic and disease-related characteristics are presented in Table 1.

**TABLE 1:** Demographic characteristics of patients with CHF (*n* = 324).

Variable	Category	n/Mean	%/SD
Gender			
	Male	209	64.5
	Female	115	35.5
Education			
	High school or below	281	86.7
	Associate’s degree or above	43	13.3
Health insurance			
	Without	103	31.8
	With	221	68.2
Marriage			
	Married	251	77.5
	Single/divorced/ widowed	73	22.5
Comorbidity of chronic diseases			
	Without	100	30.9
	With	224	69.1
Disease course			
	1 year or less	96	29.6
	1–3 years	116	35.8
	3 years or more	112	34.6
NYHA classification			
	II-class	108	33.3
	III-class	107	33.0
	IV-class	109	33.6
LVEF (%)		46.04	13.35
eGFR		62.47	25.30
Depression score		7.16	3.25
Insomnia score		13.40	5.35
home volume management score		93.35	14.35

### 3.3 Comparison of clinical indicators among patients with different NYHA functional classes

Table 2 presents the clinical indicators of patients with CHF according to NYHA functional class. Higher NYHA functional classes were associated with increased age and insomnia scores, as well as lower eGFR and home volume management scores. LVEF and depression scores appeared relatively stable across the three groups.

**TABLE 2:** Clinical indicators of patients with CHF across different NYHA functional classe.

Variables	NYHA class II (*n* = 108)	NYHA class III (*n* = 107)	NYHA class IV (*n* = 109)	*F*	*P*
Age (years)	60.22 ± 11.02	65.80 ± 10.79	66.28 ± 13.10	8.97	< 0.001
LVEF (%)	45.74 ± 14.09	47.13 ± 13.37	45.26 ± 12.60	0.57	0.566
eGFR	67.48 ± 26.47	63.27 ± 25.41	56.73 ± 22.97	5.10	0.007
Depression	7.07 ± 3.37	6.89 ± 3.00	7.50 ± 3.37	1.91	0.150
Insomnia	11.76 ± 4.55	13.86 ± 5.72	14.57 ± 5.36	8.45	< 0.001
Home volume management	96.88 ± 13.81	93.93 ± 14.40	89.30 ± 13.91	8.03	< 0.001

### 3.4 Latent profile characteristics of frailty in patients with CHF

**FIGURE 1:**
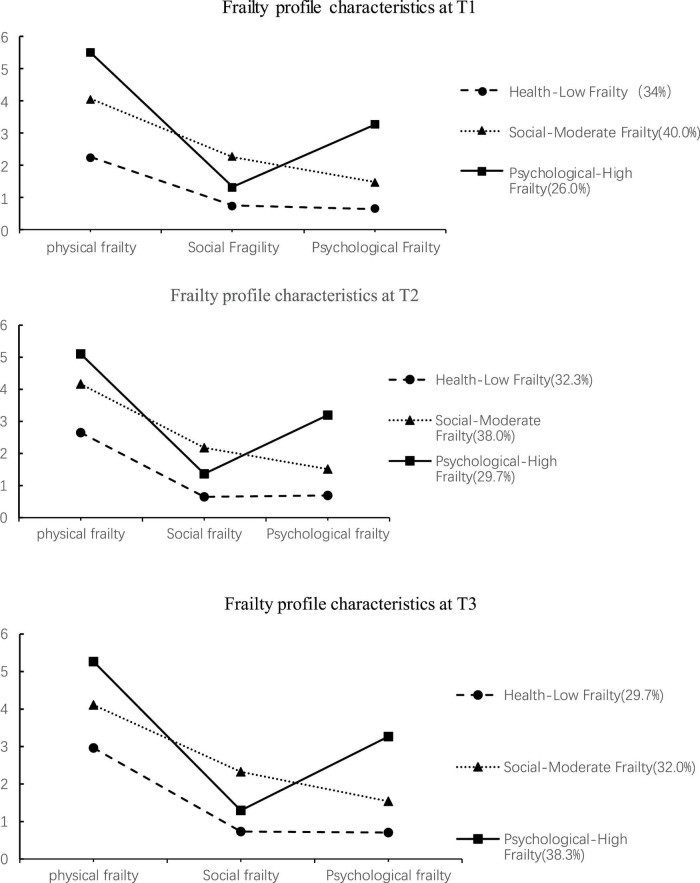
Profile of frailty subtypes in CHF patients at different stages.

Table 3 presents the model fit indices of latent frailty profiles among patients with CHF at different time points. The results indicated that the three-class model demonstrated good classification accuracy at T1, T2, and T3, with entropy values of 0.823, 0.829, and 0.863, respectively, all exceeding 0.80. In addition, both the Lo–Mendell–Rubin likelihood ratio test (LMR) and bootstrap likelihood ratio test (BLRT) were statistically significant (all *P* < 0.001), suggesting that the three-class model provided a better fit than the two-class model. Although the four-class model yielded lower AIC, BIC, and adjusted BIC values, it did not substantially improve entropy, and some subclasses showed limited discrimination and clinical interpretability. Therefore, considering model fit statistics, class stability, and clinical interpretability, the three-class model was selected as the optimal latent profile model at all three time points (T1, T2, and T3). Based on the profile characteristics, three latent frailty subgroups were identified: C1 (Health–Low Frailty), C2 (Social–Moderate Frailty), and C3 (Psychological–High Frailty) (Figure 1). Patients in C1 exhibited the lowest levels of impairment across the physical, psychological, and social domains; however, the proportion of this subgroup gradually declined from 34.0% at T1 to 32.3% at T2 and 29.7% at T3. Patients in C2 showed moderate physical and psychological impairment, with social dysfunction being the most prominent feature. The proportion of this subgroup decreased from 40.0% at T1 to 38.0% at T2 and 32.0% at T3. In contrast, patients in C3 experienced the greatest physical and psychological impairment, accompanied by moderate social dysfunction, and its proportion increased over time from 26.0% at T1 to 29.7% at T2 and 38.3% at T3.

**TABLE 3:** Fitting metrics for the latent profile of frailty in patients with CHF.

Stage	Model	AIC	BIC	aBIC	Entropy	LMR	BLRT	Category probability
T1	1	3212.192	3234.877	3215.845				1
	2	3088.126	3125.933	3094.214	0.764	0.004	< 0.001	66.0/34.0
	3	2989.561	3042.491	2998.085	0.823	< 0.001	<0.001	24.4/34.6/41.0
	4	2976.383	3044.436	2987.342	0.787	0.008	< 0.001	33.3/20.4/23.8/22.5
T2	1	3062.247	3084.932	3065.900				1
	2	2944.162	2981.970	2950.251	0.750	< 0.001	<0.001	31.5/68.5
	3	2838.103	2891.033	2846.627	0.829	< 0.001	<0.001	39.5/33.0/27.5
	4	2824.598	2892.651	2835.557	0.825	0.006	< 0.001	5.9/32.4/20.4/41.4
T3	1	3056.608	3079.292	3060.261				1
	2	2898.691	2936.498	2904.779	0.832	< 0.001	<0.001	59.3/40.7
	3	2788.718	2841.648	2797.242	0.863	< 0.001	<0.001	32.4/35.2/32.4
	4	2777.495	2845.548	2788.454	0.817	0.003	0.013	16.4/35.1/16.7/31.8

### 3.5 Distribution of frailty status and latent frailty profiles across NYHA functional classes

Supplementary Table 1 show the distribution of frailty status and latent frailty profiles across different NYHA functional classes. The prevalence of frailty increased progressively with worsening NYHA class, rising from 62.0% in patients with NYHA class II to 85.3% in those with NYHA class IV, whereas the proportion of non-frail patients decreased accordingly. In terms of latent profiles, the proportion of patients in C1 (Health–Low Frailty) declined with increasing NYHA class, while the proportions of C2 (Social–Moderate Frailty) and C3 (Psychological–High Frailty) increased. Patients with NYHA class II were predominantly classified into C1 (49.1%), whereas those with NYHA class III and IV were mainly assigned to C2 (42.1 and 49.5%, respectively).

### 3.6 Probability of potential transitions among frailty subtypes in patients with CHF

Table 4 presents the latent transition analysis (LTA) results. During the T1–T2 interval, the probabilities of remaining in the same latent profile were 63.9, 56.8, and 59.4% for C1 (Health–Low Frailty), C2 (Social–Moderate Frailty), and C3 (Psychological–High Frailty), respectively, indicating bidirectional transitions among all three profiles. During the T2–T3 interval, the probability of remaining in C1 increased to 69.2%, with no transitions to C2. The probability of remaining in C2 was 44.2%, while transition probabilities from C2 to C1 and C3 were 21.9 and 34.0%, respectively. For C3, the probability of remaining in the same profile increased to 76.5%, and no transitions from C3 to C1 were observed.

**TABLE 4:** Probability matrix for potential category transitions in frailty among CHF patients (*n* = 324).

Project	Health-low frailty	Social-moderate frailty	Psychological-high frailty
T1∼T2			
Health-low frailty	63.9%	26.7%	9.4%
Social-moderate frailty	18.8%	56.8%	24.4%
Psychological-high frailty	9.7%	30.9%	59.4%
T2∼T3			
Health-low frailty	69.2%	30.8%	0.0%
Social-moderate frailty	21.9%	44.2%	34.0%
Psychological-high frailty	0.0%	23.5%	76.5%

### 3.7 Factors influencing transitions between frailty subtypes

The coding of independent variables is presented in Supplementary Table 2. The results of the multivariable logistic regression analysis are shown in Table 5. Age, health insurance status, NYHA functional class, estimated glomerular filtration rate (eGFR), depression, insomnia, and home-based volume management were identified as significant factors associated with transitions between frailty profiles in patients with CHF. Overall, older age, lack of health insurance coverage, poorer cardiac function, lower eGFR, more severe insomnia, and poorer home-based volume management were associated with transitions toward more severe frailty profiles. In contrast, better home-based volume management was associated with maintaining or transitioning to less severe frailty profiles.

**TABLE 5:** Odds ratios for transitions between frailty classes among patients with CHF under the influence of covariates (*n* = 324).

Variable		T1ria			T2ria		
		C1 (odds ratio)	C2 (odds ratio)	C3 (odds ratio)	C1 (odds ratio)	C2 (odds ratio)	C3 (odds ratio)
Age	C1	REF	1.040	1.015	REF	0.959	/
	C2	0.946	REF	1.011	0.975	REF	1.041
	C3	/	0.922*	REF	/	0.991	REF
Gender (male vs. female)	C1	REF	2.357	2.319	REF	2.391	/
	C2	0.875	REF	0.440	1.702	REF	1.803
	C3	/	0.684	REF	/	0.667	REF
Marital (single/divorced/widowed vs. married)	C1	REF	0.932	2.170	REF	0.782	/
	C2	0.128	REF	3.960	1.734	REF	0.573
	C3	/	0.633	REF	/	0.932	REF
Health insurance (without vs. with)	C1	REF	2.414	2.990	REF	2.802	/
	C2	0.161	REF	4.975**	0.752	REF	4.729**
	C3	/	0.310	REF	/	0.262	REF
Disease course (≤ 1 year vs. ≥ 3 years)	C1	REF	1.444	0.573	REF	0.245	/
	C2	0.640	REF	0.791	1.988	REF	0.976
	C3	/	0.462	REF	/	0.719	REF
Disease course (1–3 years vs. ≥ 3 years)	C1	REF	1.013	0.441	REF	0.408	/
	C2	0.319	REF	0.721	0.947	REF	1.054
	C3	/	0.423	REF	/	0.439	REF
Comorbidity of chronic diseases (without vs. with)	C1	REF	1.097	0.380	REF	2.507	/
	C2	0.457	REF	0.516	2.650	REF	0.472
	C3	/	1.295	REF	/	1.586	REF
Education (high school or below vs. Associate’s degree or above)	C1	REF	1.929	1.194	REF	0.816	/
	C2	0.651	REF	0.247	0.687	REF	0.341
	C3	/	0.590	REF	/	2.416	REF
NYHA (Class II vs. Class IV)	C1	REF	0.800	0.239	REF	0.024**	/
	C2	1.777	REF	1.852	2.699	REF	0.126*
	C3	/	2.262	REF	/	2.359	REF
NYHA (Class III vs. Class IV)	C1	REF	0.926	0.689	REF	0.116	/
	C2	1.953	REF	1.223	2.005	REF	0.677
	C3	/	2.264	REF	/	1.285	REF
eGFR	C1	REF	0.973*	0.994	REF	0.968	/
	C2	0.984	REF	0.959**	1.012	REF	0.986
	C3	/	0.984	REF	/	0.973	REF
LVEF	C1	REF	1.003	0.952	REF	1.018	/
	C2	0.948	REF	1.019	0.986	REF	0.993
	C3	/	0.979	REF	/	0.979	REF
Insomnia	C1	REF	1.064	1.314*	REF	1.549**	/
	C2	0.698*	REF	1.079	0.943	REF	1.040
	C3	/	0.958	REF	/	1.016	REF
Depression	C1	REF	1.112	1.263	REF	0.958	/
	C2	0.829	REF	0.919	0.909	REF	0.844*
	C3	/	0.912	REF	/	0.886	REF
Home Volume Management	C1	REF	0.971	0.903*	REF	0.918**	/
	C2	1.186**	REF	0.968	1.046*	REF	0.991
	C3	/	1.034	REF	/	1.130**	REF

*p < 0.05,

**p < 0.01. C1, Healthy-low frailty; C2, Social-moderate frailty; C3, Psychological-high frailty. REF refers to the reference category where participants remained in their original group; / indicates that very few participants, or none, changed frailty subtypes.

## 4 Discussion

### 4.1 Frailty is highly prevalent in patients with CHF and closely associated with cardiac functional status

Frailty is a common geriatric syndrome in patients with chronic heart failure (CHF) and is closely associated with disease progression and adverse clinical outcomes. In the present study, the prevalence of frailty was high among patients with CHF and increased progressively with worsening NYHA functional class, suggesting a close relationship between impaired cardiac function and frailty severity. Previous studies have shown that prolonged low cardiac output in CHF can reduce physiological reserve through mechanisms including chronic inflammation, neurohormonal dysregulation, and skeletal muscle wasting, thereby contributing to the development and progression of frailty. In turn, frailty may further impair exercise tolerance, self-management capacity, and resilience to stressors, leading to an increased risk of rehospitalization and mortality (27, 28). Therefore, frailty and CHF may form a vicious cycle that accelerates disease deterioration, making frailty an important indicator of overall functional status and long-term prognosis in patients with CHF.

In the present study, the prevalence of frailty among patients with CHF was high and showed a worsening trend over the follow-up period. On the one hand, the study population was relatively old and commonly affected by multiple chronic conditions, which may have contributed to the accumulation of frailty deficits (29). On the other hand, the unequal distribution of healthcare resources and limited access to continuous rehabilitation services in some regions of Xinjiang, China, may have hindered functional recovery after discharge and further accelerated frailty progression (30). Analysis by NYHA functional class further revealed that patients with more advanced cardiac dysfunction were more likely to exhibit moderate or high levels of frailty. This finding is consistent with previous studies (31), suggesting that worsening cardiac function may increase the risk of multidimensional frailty by impairing not only physical functioning but also psychological and social wellbeing. Furthermore, three relatively stable frailty subgroups were identified across all time points, namely Health–Low Frailty, Social–Moderate Frailty, and Psychological–High Frailty. This finding is similar to that reported by Li et al. (6) in community-dwelling older adults, indicating that frailty has a relatively stable heterogeneous structure across populations. However, unlike the general older population, frailty in patients with CHF is influenced not only by age-related functional decline but also by disease-specific factors associated with cardiac dysfunction. Therefore, the identified frailty subgroups reflect not only differences in frailty severity but also distinct patterns of multidimensional functional impairment. Patients in the Health–Low Frailty subgroup exhibited relatively mild frailty symptoms and generally maintained better self-care ability and overall functional status (32). In contrast, patients in the Social–Moderate Frailty subgroup were characterized by more pronounced social dysfunction, which may be related to disease-related activity limitations, reduced social participation, and impaired role functioning (33). Notably, the proportion of patients in the Psychological–High Frailty subgroup increased over time, suggesting that psychological impairment may represent an important feature of frailty progression in CHF. Previous studies have similarly reported that long-term disease burden and functional limitations can increase psychological distress, reduce social engagement, and ultimately exacerbate frailty (34).

### 4.2 Subgroup transitions of frailty in patients with chronic heart failure

Frailty is a dynamic syndrome, and transitions between its latent classes reflect the progressive depletion of physiological reserve in patients with chronic heart failure (CHF). In this study, the Healthy-low frailty subgroup exhibited relatively limited stability, indicating that even patients with low frailty may still carry potential vulnerability in the context of living with chronic disease. This suggests that early risk identification and continuous follow-up management should be prioritized even for patients in this subgroup in clinical practice. The Social-moderate frailty subgroup showed distinct bidirectional transition patterns, with some patients reverting to the Healthy-low frailty state and others progressing to the Psychological-high frailty state. This indicates that this moderate frailty subgroup represents a reversible yet unstable transitional phase, which may serve as a critical window for intervention. Strengthening symptom management, exercise rehabilitation, and social support during this period may help promote frailty reversal and slow disease progression (6). In contrast, the Psychological-high frailty subgroup demonstrated greater persistence and cumulative characteristics, with only a small proportion of patients able to revert to the Healthy-low frailty state. This finding suggests that once high frailty develops, patients often experience increased psychological burden and reduced self-management capacity, making recovery difficult in the short term. Over the course of the disease, this state may gradually become relatively fixed (35). These results highlight the need for comprehensive, multi-dimensional management of patients in this high-risk subgroup in clinical settings to delay frailty progression and improve long-term outcomes.

### 4.3 Factors associated with transitions between frailty subgroups in patients with CHF

The present study found that increasing age reduced the likelihood of transitioning from the Psychological-high frailty subgroup to the Social-moderate frailty subgroup, indicating poor reversibility of frailty in older patients. Previous studies (36) have similarly identified advanced age as a key predictor of persistent frailty, readmission, and adverse outcomes in patients with chronic heart failure. In addition, age-related declines in physical activity, nutritional status, and adaptive capacity (37) may further limit the reversibility of frailty states.

Patients without medical insurance were more likely to transition into the Psychological-high frailty subgroup. Li et al. (38) found that medical insurance buffers the risk of depression among patients with chronic diseases, while the lack of insurance may exacerbate patients’ psychological burden and disease vulnerability. Within the context of China’s healthcare system, the absence of medical insurance not only means higher direct medical costs for patients but may also reduce their willingness to attend outpatient follow-ups, maintain continuous medication use, and participate in community follow-up care, thereby weakening the effectiveness of continuous chronic disease management (39). Meanwhile, disparities remain in the implementation of guideline-directed therapies across different regions and levels of healthcare institutions in China, suggesting that healthcare resource accessibility remains an important factor influencing long-term management quality.

The New York Heart Association (NYHA) functional classification is an important indicator reflecting the severity of heart failure. The present study showed that patients with NYHA class II were less likely to transition to the moderate and high frailty subgroups compared with those in NYHA class IV, indicating that better cardiac function helps maintain a low frailty level. Consistently, Vitale et al. (10) reported that higher NYHA class is associated with more severe functional limitation and reduced exercise capacity, thereby promoting the development of multidimensional frailty. Although the strength of the association between NYHA class and frailty varies across studies, patients with higher NYHA class are generally more prone to multi-domain frailty and adverse outcomes (40).

A higher estimated glomerular filtration rate (eGFR) reduced the risk of transition to more severe frailty subgroups, suggesting that preserved renal function exerts a protective effect against frailty progression. Heart failure and chronic kidney disease frequently coexist and contribute to cardiorenal syndrome, with the two conditions interacting via inflammatory activation, volume overload, metabolic disorders and other mechanisms (41). Accumulating evidence has demonstrated that reduced eGFR is closely correlated with elevated inflammatory factors, and chronic inflammation is recognized as a key biological mechanism underlying frailty (42). Furthermore, impaired renal function is linked to increased risks of hospitalization and mortality, indicating its involvement in frailty deterioration and adverse clinical outcomes among patients with chronic heart failure (43).

This study found that insomnia increased the risk of transitioning to more severe frailty subgroups and reduced the probability of recovery to the low frailty subgroup, which is consistent with the findings of Zheng et al. (44). Sleep disorders are prevalent among patients with chronic heart failure. They may continuously deplete bodily reserve capacity by activating the sympathetic nervous system, exacerbating inflammatory responses and disrupting endocrine function, thereby hindering the improvement of frailty (45). Zheng et al. (44) also indicated that insufficient sleep aggravates daytime fatigue and impairs patients’ ability to engage in exercise rehabilitation and self-management, creating a vicious cycle. Accordingly, regular sleep assessment and early identification of persistent sleep disturbances should be emphasized for CHF patients. Non-pharmacological interventions including relaxation therapy, mindfulness training and lifestyle modification (46) can be implemented to restore normal sleep patterns and improve sleep quality.

This study revealed that depressive symptoms elevated the probability of remaining in the Social-moderate frailty subgroup among CHF patients, yet did not significantly facilitate their progression to the Psychological-high frailty subgroup. Depressive status is typically characterized by decreased interest, social withdrawal, and reduced behavioral initiative. Patients tend to actively reduce interactions with relatives, friends and social circles, ultimately leading to insufficient social support and declined social participation (47). A systematic review (2) demonstrated that depression serves as a critical component of psychological and social frailty, and exacerbates patients’ vulnerability by impairing emotional status and self-management capacity. Therefore, for CHF patients with depressive symptoms, clinical practitioners should not only focus on emotional disturbances, but also attach importance to impaired social function, including weakened social support systems and reduced social engagement.

Volume management is essential to maintaining circulatory stability in CHF patients. A higher level of capacity management significantly reduces the risk of progression to the Social-moderate frailty and Psychological-high frailty subgroups, and facilitates patients’ transition toward the Healthy-low frailty subgroup. Volume management improves patients’ exercise tolerance and preserves daily function by alleviating volume overload, optimizing tissue perfusion and relieving edema (48). As a long-term self-management behavior, it encompasses core components such as low-sodium diet, daily weight monitoring, and early identification of volume overload (26). Previous studies (48) have verified that effective volume management can mitigate volume overload and stabilize circulatory conditions. Accordingly, medical staff should incorporate standardized volume management into frailty risk control strategies for heart failure patients. Targeted health education for patients and caregivers should be strengthened to improve the early identification of volume imbalance, so as to prevent the deterioration of frailty status.

## 5 Conclusion

CHF patients presented an overall elevated frailty level. Three stable frailty subgroups were identified across all time points, namely Healthy-low frailty, Social-moderate frailty, and Psychological-high frailty. Subgroup transitions were observed during the follow-up period, demonstrating the dynamic and stage-dependent nature of frailty. Given the distinct characteristics of each frailty phenotype, stratified management strategies should be implemented clinically. For patients with Healthy-low frailty, regular disease monitoring and targeted health guidance are recommended. For those with Social-moderate frailty, interventions should prioritize optimizing social support and enhancing home-based self-management. For individuals in the Psychological-high frailty subgroup, greater emphasis should be placed on psychological counseling, sleep regulation, and comprehensive symptom control. Additionally, dynamic multi-dimensional frailty assessment and long-term follow-up are warranted, especially for older patients and those with poor cardiac or renal function, to slow frailty progression and optimize long-term clinical prognoses.

## 6 Limitations

This study has several limitations. First, although this study adopted a longitudinal design, the relatively limited sample size and single-center observational nature restricted causal inference and the generalizability of the findings. Second, several potential confounding variables were not included in the analytical model, which may affect the estimation results. Furthermore, several indicators were collected based on participants’ self-reports, which may lead to recall bias and measurement errors. Future studies should adopt a multicenter design with a larger sample size to improve representativeness and generalizability. Additionally, more confounding factors should be incorporated, and objective indicators should be combined to achieve comprehensive evaluation. If conditions permit, interventional studies are warranted to further clarify causal relationships.

## Data Availability

The raw data supporting the conclusions of this article will be made available by the authors, without undue reservation.
