# Supplementary material for: Identification of frailty heterogeneity and its transition trajectories in patients with chronic heart failure: a latent transition analysis
Source: Front Med (Lausanne). 2026 Jul 2;13:1862183. doi: 10.3389/fmed.2026.1862183 (PMC13372607; doi:10.3389/fmed.2026.1862183)
Supplement: Supplementary file 1 [file Supplementary_file_1.docx]

Supplementary Figure 1 Completion of follow-up among study participants
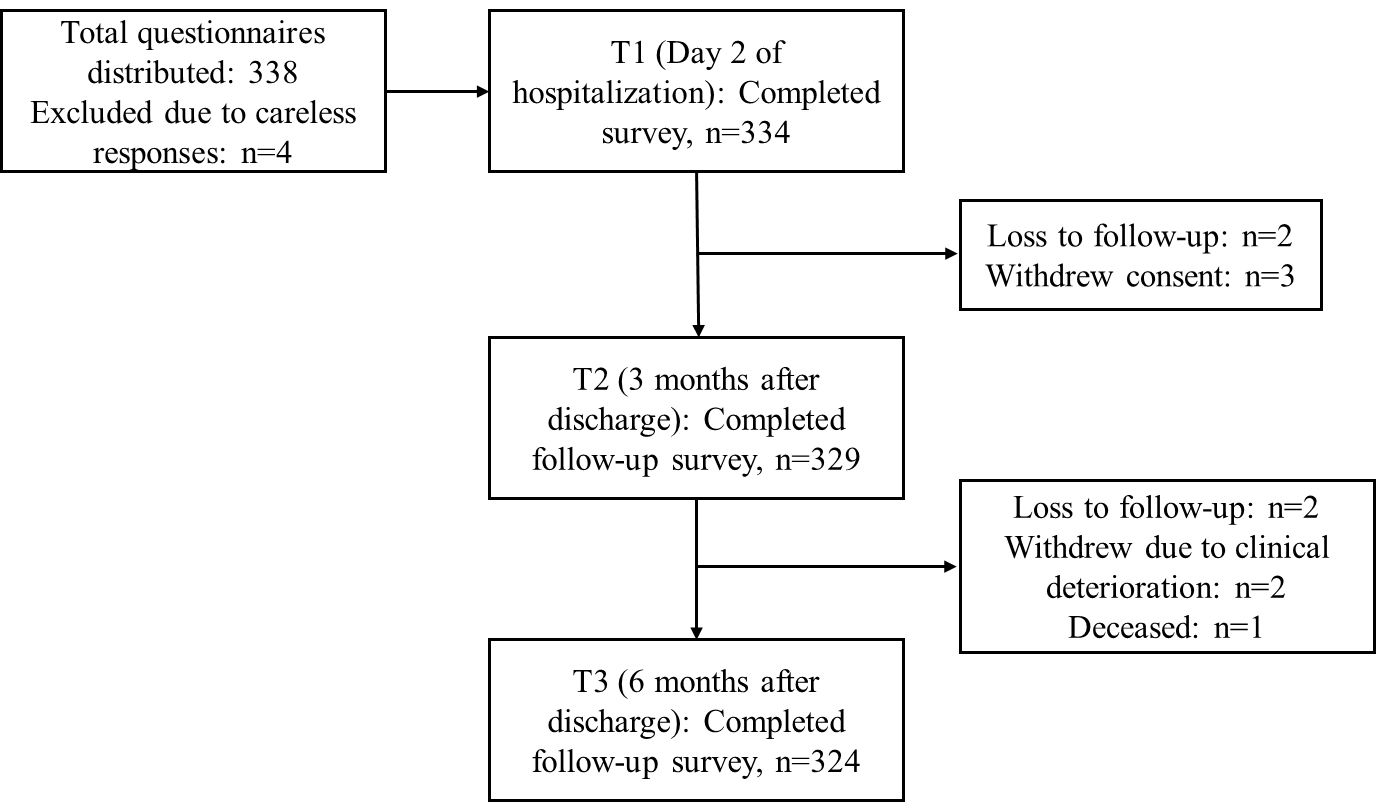


Supplementary Table 1. Distribution of Frailty Status and Latent Frailty Profiles Across NYHA Functional Classes at Baseline (n=324)

Supplementary Table 2. Method of Assigning Covariates

| Covariates: | Assignment method |
| --- | --- |
| Gender | 1 = Male, 2 = Female (Reference) |
| Health insurance | 0 = without, 1 = with (Reference) |
| Marital | 1 = Married, 2 = Unmarried/Divorced/Widowed (Reference) |
| Education | 1 = High school or below, 2 = Associate's degree or above (Reference) |
| Comorbidity of chronic diseases | 0 = without, 1 = with (Reference) |
| Disease course | 1 = 1 year or less, 2 = 1–3 years, 3 =3 years or more (Reference) |
| NYHA classification | II-class = 1, III-class = 2, IV-class = 3 (Reference) |
| LVEF | Substitute original values |
| eGFR | Substitute original values |
| Age | Substitute original values |
| Insomnia score | Substitute original values |
| Depression score | Substitute original values |
| Hom volume management score | Substitute original values |
